# Discovery of Geranylgeranyl Pyrophosphate Synthase (GGPPS) Paralogs from *Haematococcus pluvialis* Based on Iso-Seq Analysis and Their Function on Astaxanthin Biosynthesis

**DOI:** 10.3390/md17120696

**Published:** 2019-12-12

**Authors:** Danqiong Huang, Wenfu Liu, Anguo Li, Chaogang Wang, Zhangli Hu

**Affiliations:** 1 Shenzhen Key Laboratory of Marine Bioresource and Eco-environmental Science, Shenzhen Engineering Laboratory for Marine Algal Biotechnology, Guangdong Provincial Key Laboratory for Plant Epigenetics, College of Life Sciences and Oceanography, Shenzhen University, Shenzhen 518060, China; dqhuang@szu.edu.cn (D.H.); liuwenfu2017@email.szu.edu.cn (W.L.); 1800251012@email.szu.edu.cn (A.L.); 2 Key Laboratory of Optoelectronic Devices and Systems of Ministry of Education and Guangdong Province, College of Optoelectronic Engineering, Shenzhen University, Shenzhen 518060, China

**Keywords:** *GGPPS*, *Haematococcus pluvialis*, astaxanthin, Iso-Seq

## Abstract

*Haematococcus pluvialis* is widely distributed in the world and well known as the richest natural source of astaxanthin that is a strong antioxidant with excellent commercial value. The pathway of astaxanthin biosynthesis in *H. pluvialis* has been documented as an enzymatic reaction. Several enzymes have been reported, but their isoforms or homologs have not been investigated genome-wide. To better understand the astaxanthin biosynthesis pathway in *H. pluvialis*, eight candidates of the geranylgeranyl pyrophosphate synthase gene (*HpGGPPS*) predicted from Iso-seq data were isolated in this study. The length of coding region of these candidates varied from 960 bp to 1272 bp, composing of 7–9 exons. The putative amino acids of all candidates composed the signature domain of *GGPPS* gene. However, the motifs in the domain region are varied, indicating different bio-functions. Phylogenetic analysis revealed eight candidates can be clustered into three groups. Only two candidates in Group1 encode the synthase participating in the astaxanthin formation. The yield of astaxanthin from these two candidates, 7.1 mg/g (DW) and 6.5 mg/g (DW) respectively, is significant higher than that from *CrtE* (2.4 mg/g DW), a *GGPPS* gene from *Pantoea ananatis*. This study provides a potential productive pathway for astaxanthin synthesis.

## 1. Introduction

Astaxanthin (C_40_H_52_O_4_), a keto-carotenoid, has been well known as super vitamin E due to its hydroxyl and ketone functional groups and multiple conjugated double bonds that can reduce the reactive oxidizing molecule. Thus, astaxanthin can be consumed as a dietary supplement or aging related-cosmetics. Meanwhile, astaxanthin also has been used as an aquaculture consumption due to its additive of the blood-red color, contributing to over $500 million a year in the market [1]. Astaxanthin can be produced chemically in the factory or naturally from organisms such as the microalga *Haematococcus pluvialis* and the yeast fungus *Xanthophyllomyces dendrorhous* [2]. Compared with chemically synthetic astaxanthin from the Wittig reaction using asta-C15-triarylphosphonium salt and the C10-dialdehyde [3], the naturally isolated astaxanthin has 20-times higher antioxidant activity and is capable for human consumption [4,5].

As the most important source of natural astaxanthin, it is imperative to understand the biological process and the regulation of astaxanthin formation in *H. pluvialis* aiming to improve the productivity of astaxanthin. The pathway of astaxanthin production in *H. pluvialis* is complex but still has been described, which is originally started from pyruvate and glyceraldehyde-3-phosphate (GA-3P) followed by a series of enzymatic reactions [6,7]. Briefly, isopentenyl diphosphate (IPP), the precursor of carotenoids, was synthesized through methyl-d-erythritol 4-phosphate (MEP) pathway using pyruvate, GA-3P, and enzymes of DXS and IspC-IspH [8,9]. Subsequently, IPP and dimethylallyl diphosphate (DMAPP, an isomer of IPP that was formed by isopentenyl pyrophosphate isomerase (IPI) or 4-hydroxy-3-methylbut-2-enyl diphosphate reductase (HDR)), were used to form a C20 compound, geranylgeranyl pyrophosphate (GGPP), with the help of geranylgeranyl pyrophosphate synthase (GGPPS) [10,11,12,13]. Carotenoids were then initialized using GGPP, in the main order of phytoene, ζ-carotene, lycopene, β-carotene, cathaxanthin, and astaxanthin, with cooperation of enzymes of phytoenesysthase (PSY), phytoenedesaturase (PDS), ζ-carotenedesaturase (ZDS), lycopene β-cyclase (LCY-b), β-carotene ketolase (BKT), and β-carotene 3,3′-hydroxylase (CrtR-b) [2,7,13]. Thereby, the GGPPS, a key enzyme defining the initial GGPP availability, is crucial for the subsequent flux of carotenoids biosynthesis, theoretically. It has been reported that the mechanism of the enzymatic reaction converting IPP to GGPP is conserved among organisms from bacteria to human [14,15]. However, the number of *GGPPS* paralogs were varied within species, as high as 12 in *Arabidopsis* [16] and as low as one in *Chlamydomonas reinhardtii* [17]. Specifically, three *GGPPS* genes were found in *H. pluvialis* based on a RNA-Seq transcriptome analysis (Genbank accessions: KX236181–KX236183). With the advantage of obtaining the full-length of transcripts to identify isoforms, alternative splicing, fusion transcripts, LncRNA, and new homologous genes, the single-molecule real-time (SMRT) long-read isoform sequencing (Iso-Seq) developed by Pacific BioSciences (PaciBio) is more preferred to discover more paralogs over the RNA-Seq technology [18]. As one of successful examples, more than 113,000 novel splice isoforms were discovered in a Iso-Seq data of *Sorghum bicolor* L. Moench and some of them are functional which confirmed by additional experiments of gene cloning and RT-PCR [19].

Since the whole genome sequence of *H. pluvialis* is available [20], it is possible to discover more reliable novel *GGPPS* genes from the Iso-Seq data. The main objectives of this study were to explore more *GGPPS* paralogs in *H. pluvialis* using an Iso-Seq data, to determine their evolutionary relationship, and to characterize their performance on astaxanthin biosynthesis. By comparing the productivity of astaxanthin using different *GGPPS* genes from *H. pluvialis*, it is expected to arrange a more efficient pathway for astaxanthin biosynthesis.

## 2. Results

### 2.1. Cloning and Sequence Characterization of HpGGPPS Genes

Using three reported *GGPPS* genes of *H. pluvialis* retrieved from Genbank (accession nos. KX236181, KX236182, and KX236183), eight candidates were carried out from the Iso-Seq data by BlastN analysis with e-value of 0. Using designated gene-specific primers, the full-length coding sequences (CDS) of eight putative *HpGGPPS* genes (*HpGGPPS1-1*, *HpGGPPS1-2*, *HpGGPPS2-1*, *HpGGPPS2-1*, *HpGGPPS3-1*, *HpGGPPS3-2*, *HpGGPPS3-3*, and *HpGGPPS3-4*) were successfully isolated. Sequences were submitted to Genbank with accession numbers MN689792–MN689799.

The nucleotide sequence analysis revealed that eight putative *HpGGPPS* genes are ranging 1011–1455 bp in length and varied from 960 bp to 1272 bp in CDS region (Supplemental Table S1). Their deduced amino acids are ranging 319–423 aa with putative molecular weight of 36.27–45.75 KDa, respectively (Supplemental Table S1). The alignment of nucleotide sequences of eight *HpGGPPS* genes suggested 39.9–100% similarity in the CDS region (Supplemental Figure S1). Clearly, three classes were presented according to the similarity. High nucleotide sequence similarity was found within classes, including 98.6% identity between *HpGGPPS1-*1 and *HpGGPPS1-2*, 97.4% identity between *HpGGPPS2-1* and *HpGGPPS2-2*, and 99.1–100% identity among *HpGGPPS3-1*, *HpGGPPS3-2*, *HpGGPPS3-3*, and *HpGGPPS3-4*. It is noted that In-Del variation was found between sequences of *HpGGPPS3-1* and *HpGGPPS3-2* (sharing 100% similarity with each other), and between sequences of *HpGGPPS3-3* and *HpGGPPS3-4* (sharing 100% similarity with each other). By contrast, low percentage of nucleotides identity was found among classes, which was lower than 49%.

**Figure 1:**
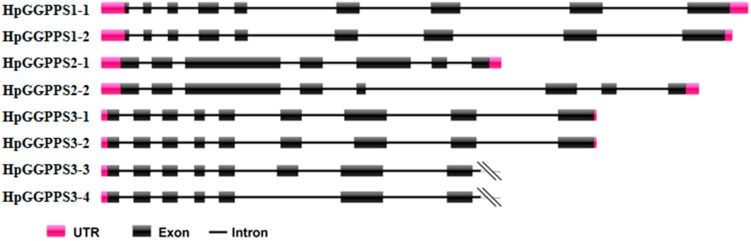
Schematic diagram of *HpGGPPS* gene structure, which was predicted by aligning with corresponding genome sequences. The slash indicates incomplete genome sequences resulting partial gene structure.

By the local BlastN against *H. pluvialis* genome sequences, *HpGGPPS1-1*, *HpGGPPS1-2*, *HpGGPPS2-1*, and *HpGGPPS2-2* hit different scaffolds, revealing different genome region. Differently, *HpGGPPS3-1* and *HpGGPPS3-2* hit the same scaffold, as well as *HpGGPPS3-3* and *HpGGPPS3-4*. By considering their 100% sequence identity, they might be isoforms from alternative splicing. Further sequences alignment of *HpGGPPS* genes with corresponding genome scaffolds confirmed that *HpGGPP3-1* and *HpGGPP3-2* were isoforms from modified splicing of intron, while *HpGGPPS3-3* and *HpGGPPS3-4* were isoforms from exon exclusion (Figure 1). Gene structure analysis suggested that *HpGGPPS1-1* and *HpGGPPS1-2* were composed from nine exons, as well as *HpGGPPS3-1*, and *HpGGPPS3-2*. Diversely, *HpGGPPS2-1* and *HpGGPPS2-2* were from seven and eight exons, respectively (Figure 1). Due to the missing nucleotide information in the scaffold, the gene structure of *HpGGPPS3-3* and *HpGGPPS3-4* is not complete.

### 2.2. Molecular Evolution of HpGGPPS Genes

**Figure 2:**
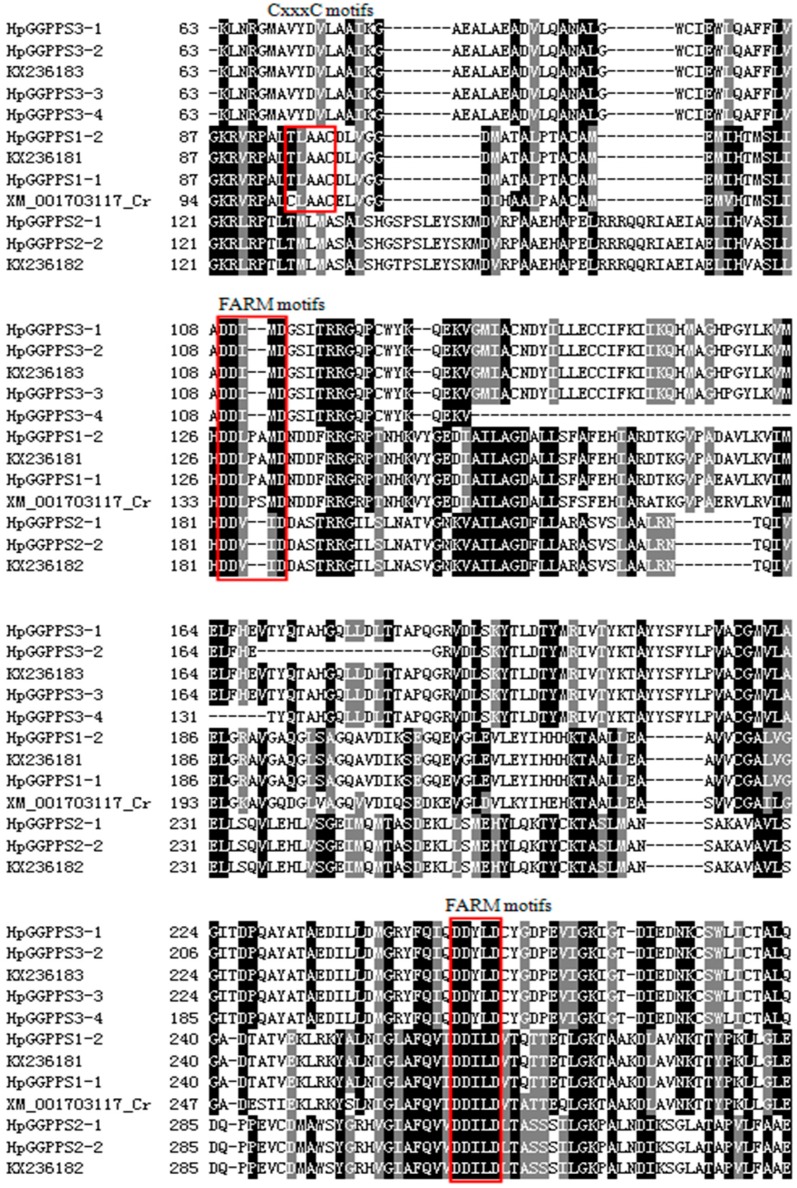
Alignment of Polyprenyl_syn domain region in the deduced amino acids of *GGPPS* genes. The motifs for CxxxC, FARM, and SARM are red squared. The sequence of XM_001713117 is from *Chlamydomonas reinhardtii*, while the rest of 11 sequences are from *Haematococcus pluvialis*.

To predict the function of isolated *HpGGPPS* candidates, the domain of their putative amino acids was analyzed. According to SMART online prediction, the deduced amino acids of seven out of eight candidates contained the full domain of Polyprenyl_syn (Pfam accession: PF00348), which is associated with isoprenoid compounds synthesis. The excluded candidate, *HpGGPPS3-4*, has incomplete Polyprenyl_syn domain, because of the missing exon in comparison with *HpGGPPS3-3*. By aligning the deduced amino acids of eight *HpGGPPS* candidates from this study and four from Genbank (KX236181, KX236182, and KX236183 from *H. pluvialis*; XM001703117 from *Chlamydomonas reinhardtii*), motifs analysis was performed and results revealed that all 12 genes contained the signature asparate rich motifs, SARM (DDxxxD, which x refers to any amino acids) and FARM (DDxxxxD, which x refers to any amino acids), commonly found in *GGPPS* gene (Figure 2). Unlike the SARM domain conserved among aligned genes, the variation was found in the FARM motifs. In detailed, *HpGGPPS1-1*, *HpGGPPS2-1*, KX236181, and XM_001703117 have the traditional motifs of DDxxxxD, while rest genes have a modified motifs of DDxx--D. Moreover, *HpGGPPS1-1*, *HpGGPPS2-1*, KX236181, and XM_001703117 have the additional CxxxC motifs, indicating they might have different bio-functions from the rest of genes. Besides, *GGPPS* genes from *H. pluvialis* (*HpGGPPS1-1*, *HpGGPPS2-1*, and KX236181) have the modified TxxxC motifs, comparing with the *GGPPS* gene from *C. reinhardtii*.

**Figure 3:**
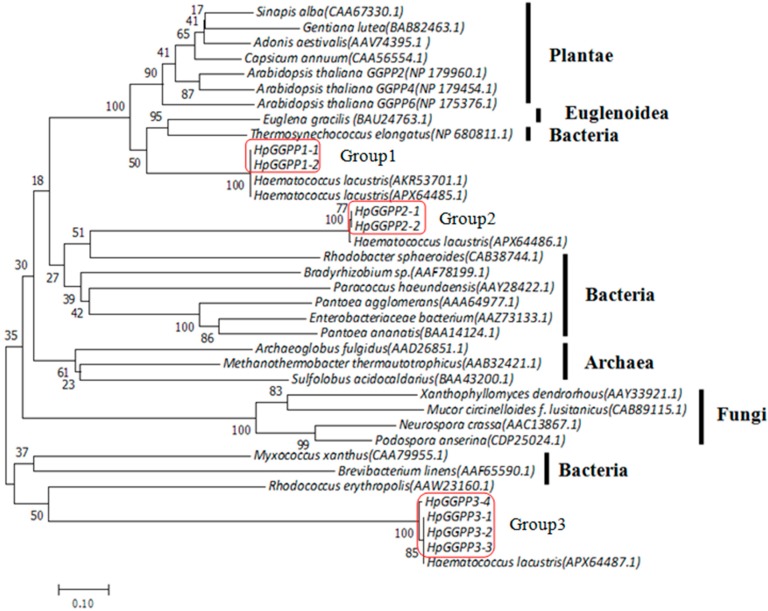
Phylogenetic relationships of the deduced amino acid sequences of *GGPPS* genes obtained in this study and retried from Genbank. Numbers in parentheses are accession numbers of *GGPPS* genes. The phylogenetic tree was constructed by MEGA version 7.0 using neighbor-joining method. Bootstrap values from the percentages of 1000 replications are indicated beside each node.

To further evaluate the evolutionary pattern of *HpGGPPS* genes, a phylogenetic tree was constructed using *GGPPS* genes obtained in this study and retrieved from Genbank which originally from other species including Planta, Bacteria, Archaea, and Fungi (Figure 3). Eight *HpGGPPS* genes were clustered into three distinguish groups, paralleled with the three classes identified according to sequence similarity. The Group1 genes, composing of *HpGGPPS1-1* and *HpGGPPS1-2*, were clustered with *GGPPS* genes from Plantae and were more close to *Euglena gracillis* and *Thermosynehococcus elongatus* belonging to photosynthetic unicellular species, compared with other high plant species. The Group2 genes, composing of *HpGGPPS2-1* and *HpGGPPS2-2*, were in a distinct cluster apart from the cluster containing *HpGGPPS3-1*, *HpGGPPS3-2*, *HpGGPPS3-3*, and *HpGGPPS3-4* (Group3). It is interesting that six putative *HpGGPPS* genes from Group2 and Group3 were more likely clustered with *GGPPS* genes from Bacteria, which implied that their function might different from *HpGGPPS1-1* and *HpGGPPS1-2* that are close to Planta evolutionarily.

### 2.3. Functional Identification of HpGGPPS Genes in Escherichia coli

**Figure 4:**
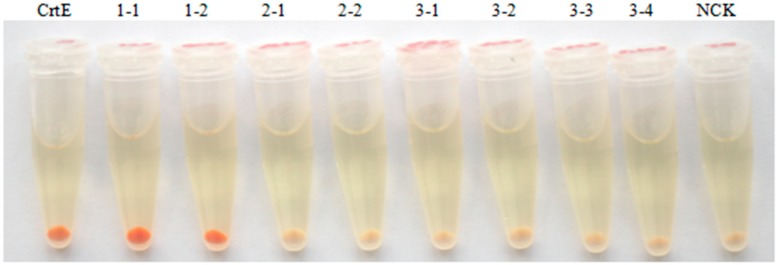
Hetero-expression experiments of pFZ153ΔE/GGPPS in *E. coli* targeting astaxanthin synthesis. *E. coli* strain BL21 (DE3) was used as the host. *CrtE* refers to the *GGPPS* gene from *Pantoea ananatis*, served as the positive control producing astaxanthin; 1-1, 1-2, 2-1, 2-2, 3-1, 3-2, 3-3, and 3-4 refers to corresponding *HpGGPPS* genes isolated in this study; NCK refers to the negative control, which is the BL21 (DE3) containing pFZ153ΔE without *GGPPS* gene for production of no astaxanthin. Colors of *E. coli* pellet are representative of at least five transformants with similar results.

The function of isolated *HpGGPPS* genes was analyzed by hetero-expression studies in *E. coli* targeting the astaxanthin formation. The transformed *E. coli* cells with pFZ153ΔE/CrtE, a plasmid containing astaxanthin biosynthetic gene cluster originally from Bacteria as the positive control, displayed orange-red color in the cell pellet as a result of pigmentation of astaxanthin (Figure 4). Served as the negative control, *E. coli* cells harboring pFZ153ΔE (NCK), a plasmid without *GGPPS* gene but with all rest genes (CrtI, CrtB, IPI, CrtY, CrtZ, CrtW) for astaxanthin production, displayed slight yellow color in the cell pellet. Similar as the positive control, the *E. coli* transformants harboring pFZ153ΔE/GGPPS1-1 (1-1) and pFZ153ΔE/GGPPS1-2 (1-2) also displayed orange-red color, but relatively darker, which might be explained by more pigmentation. By contrast, the pigmentation was not observed in *E. coli* pellets harboring pFZ153ΔE/GGPPS2-1 (2-1), pFZ153ΔE/GGPPS2-2 (2-2), pFZ153ΔE/GGPPS3-1 (3-1), pFZ153ΔE/GGPPS3-2 (3-2), pFZ153ΔE/GGPPS3-3 (3-3), or pFZ153ΔE/GGPPS3-4 (3-4) (Figure 4), indicating that they might not encode proteins associated with pigment production. Therefore, only proteins encoded by *HpGGPPS1-1* and *HpGGPPS1-2* have GGPP synthase activity to participate pigment formation.

**Figure 5:**
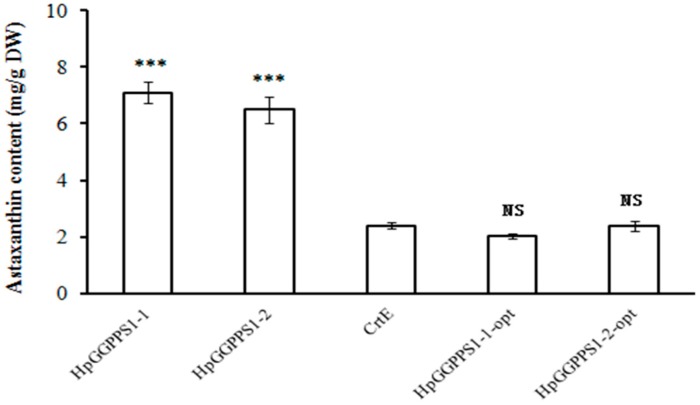
Astaxanthin content in *E. coli* (BL21 DE3) transformants with plasmids harboring astaxanthin synthetic gene cluster, according to HPLC analysis. Specifically, HpGGPPS1-1-opt and HpGGPPS1-2-opt refers to the bacteria codon optimized *HpGGPPS1-1* and *HpGGPPS1-2* gene, respectively. Data were the mean from at least three replicates and the standard error was presented as the bar. *** indicates the significant differences at the level of 0.001. NS indicates no significant differences at the level of 0.05.

To further confirm and quantify the astaxanthin production in *E. coli* cells, a HPLC analysis was successfully conducted. The chromatographic profile of astaxanthin was generated using commercial standards (Supplemental Figure S2a). As expected, an absorption peak of astaxanthin was detected in the positive control and but not detected in the negative control. Similarly, the pigment in *E. coli* cells harboring pFZ153ΔE/GGPPS1-1 and pFZ153ΔE/GGPPS1-2 have a peak at the same retention time as astaxanthin standard, indicating the existence of astaxanthin (Supplemental Figure S2b). Consisting with the color evaluation in cell pellets, astaxanthin was not detected in *E. coli* cells harboring pFZ153ΔE/GGPPS2-1, pFZ153ΔE/GGPPS2-2, pFZ153ΔE/GGPPS3-1, pFZ153ΔE/GGPPS3-2, pFZ153ΔE/GGPPS3-3, and pFZ153ΔE/GGPPS3-4. Besides, no other absorption peaks were observed in their chromatographic profile. Meanwhile, the quantity of astaxanthin was measured using a standard curve constructed from a set of astaxanthin standards. Results showed that *E. coli* cells harboring pFZ153ΔE/GGPPS1-1 produced as high as 7.1 mg astaxanthin per gram of dried cells in average from three independent pigment extraction, which is slight more (but not significant at the level of 0.05) than *E. coli* cells harboring pFZ153ΔE/GGPPS1-2 (6.5 mg/g DW in average). Notably, the positive control has significant less yield of astaxanthin (2.4 mg/g DW in average), compared with *E. coli* cells with *HpGGPPS* genes (Figure 5).

## 3. Discussion

Astaxanthin is one of the strongest antioxidants and its natural format is being demanded by increasing market looking for natural products [1]. Thereby, the topic of improving astaxanthin production from natural sources is attractive for the industry. Lots of effort has been made by scientists trying to design an efficient protocol for astaxanthin production using natural source [7]. As a major natural source of astaxanthin, *Haematococcus pluvialis* accumulated as high as 5% dry weight of astaxanthin [21] and its dairy supplements can be used human-friendly [22]. It was found that two specific enzymes, β-carotene hydroxylase (CrtR-b) and ketolase (BKT), are crucial for astaxanthin formation [23,24,25,26] and many researches were focused on aiming to improve astaxanthin content [27,28,29]. However, as the important precursor to initial the carotenoids synthesis in *H. pluvialis,* limited research was conducted for the regulation of geranylgeranyl pyrophosphate (GGPP) on astaxanthin production. It was recorded that GGPP synthase (GGPPS) plays an important role on GGPP formation [6]. In *Arabidopsis*, a well-studied species, 12 *GGPPS* genes were found and 10 of them have the ability to produce GGPP [30]. Research suggested that GGPP formation was regulated with different pattern in *Arabidopsis* [30].

Previously, there are three *GGPPS* genes from *H. pluvialis* in Genbank and one (Genbank accession no. KX236181) have the activity to catalyze GGPP formation [31]. Unfortunately, no further information was reported regarding the other two *GGPPS* genes (Genbank accession nos. KX236182, KX236183). With the expectation of discovering more paralogs having enzymatic activity, this study screened an Iso-Seq data covering transcripts from the control and stressed *H. pluvialis* cells. The stress condition was high light and salt which was normally used to induce the astaxanthin formation [20]. Eight candidates were found and their gene structures were illustrated in this study (Figure 1), using the available genome sequences (BIG Data Center GSA Database accession no. PRJCA000614) [20]. Generally, two conserved aspartate rich motifs, FARM and SARM, and flexible CxxxC motifs are presented in the domain of amino acids of *GGPPS* genes [16,31,32]. Based on the domain analysis, FARM and SARM motifs were presented in all eight *HpGGPPS* genes while CxxxC motifs was only found in *HpGGPPS1-1*, *HpGGPPS1-2*, KX236181, and XM_001703117 (Figure 2). It has been documented that FARM, SARM and CxxxC motifs are important for GGPP biosynthesis by affecting the IPP and DMAPP substrate binding process and by affecting the physical interaction between subunits of heteroimeric GPP, respectively [31,32]. Proteins with features of conserved FARM, SARM, and CxxxC motifs displayed the function of catalyzing GGPP formation [33], indicating similar function might be employed by *HpGGPPS1-1* and *HpGGPPS1-2*. Differently, the CxxxC motifs was not found in other six *HpGGPPS* genes (Figure 2). Such proteins with conserved FARM and SARM motifs but without CxxxC motifs was shown to be involved in the production of medium (C25) to long (C45) chain isoprenoids [34]. It is noted that six *HpGGPPS* genes isolated in this study (*HpGGPPS2-1*, *HpGGPPS2-2*, *HpGGPPS3-1*, *HpGGPPS3-2*, *HpGGPPS3-3*, and *HpGGPPS3-4*) and two previously reported *GGPPS* genes (KX236182 and KX236183) have modified FARM motifs (DDxx--D instead of DDxxxxD). The effect of this type of modification on their bio-function has not been reported. The feature of modified FARM motifs, conserved SARM motifs, and lacking CxxxC motifs was also observed in *GGPPS* genes from other species, such as *Linus usitatissimum*, *Ricinus communis*, *Physcomitrella patens*, *Zea mays*, and *Vitis vinifera* [16].

According to the nucleotide sequence similarity and phylogenetic tree analysis constructed from putative amino acids, eight candidates can be grouped into three classes with more than one member in each class (Figure 3). To directly evaluate their function on astaxanthin biosynthesis, hetero-expression in *E. coli* was performed using candidate genes together with other genes that are essential for astaxanthin biosynthesis. It is not surprised that two candidates, *HpGGPPS1-1* and *HpGGPPS1-2* that closely related to *GGPPS1* (Genbank accession no. KX236181), could promote the formation of astaxanthin in *E. coli* (Figure 4). Sequence similarity analysis showed that *HpGGPPS1-1* and *HpGGPPS1-2* shared 99% identity in nucleotides of coding region and only one amino acid differences. By contrast, big variation (90% identity) was found in the 5’UTR region of *HpGGPPS1-1* and *HpGGPPS1-2*, indicating there are two functional *HpGGPPS* in *H. pluvialis* rather than one as previously reported [35]. No astaxanthin or other visible pigments were observed in *E. coli* carrying the rest of six *HpGGPPS* candidates, indicating they might not directly associate with astaxanthin production. Similar findings were also found in other species, such as *Arabidposis* having *AtGGPPS5* and *AtGGPPS12* gene with GGPPS domain features but without the function of converting IPP and DMAPP to GGPP [30,36]. Additional in vitro studies could be conducted to investigate the potential function of these non-astaxanthin-related *HpGGPPS* genes.

Furthermore, the yield of astaxanthin in *E. coli* carrying *HpGGPPS1-1* and *HpGGPPS1-2* genes is about 271–296% increased than that carrying *CrtE* gene (the *GGPPS* gene driven from *Pantoea ananatis* as the positive control). In general, codon optimization is considered as an essential step to produce heterologous protein in *E. coli* to overcome the impairment of gene expression from the codon bias [37,38]. Followed by this conventional strategy, we expected more GGPPS proteins in *E. coli* transformants with codon-optimized genes, thereby leading to more astaxanthin production, theoretically. Nevertheless, *E. coli* with codon optimized *HpGGPPS1-1* and *HpGGPPS1-2* produced significant lower amount of astaxanthin (Figure 5), comparing with non-codon optimized corresponding genes, but produced similar amount of astaxanthin as *CrtE* (Figure 5). A possible explanation would be that excessive GGPPS might inhibit the astaxanthin biosynthesis in *E. coli*. This hypothesis is partly supported by the evidence that no change or downregulation of *GGPPS* was found in the salicylic acid and jasmonic acid stressed transcriptome analysis in *H. pluvialis* during astaxanthin formation [39]. Further research can be conducted to test the suppressive role of GGPPS during astaxanthin biosynthesis.

In conclusion, this study found two *HpGGPPS* gene having the synthase activity to participate in the astaxanthin biosynthesis. Non-codon optimized *H. pluvialis*-driven *GGPPS* genes produced significant higher amount of astaxanthin in *E. coli*, compared with bacteria-driven *GGPPS* gene. This study improves the understanding of the role of *GGPPS* in astaxanthin biosynthesis and thereby contributes to the design of efficient pathway for astaxanthin production.

## 4. Materials and Methods

### 4.1. Microalga Culture, Treatment, and Sample Collection

The microalga *Haematococcus pluvialis* strain 192.80, originally purchased from EPSAG (Experimental Phycology and Culture Collection of Algae, Goettingen University, Goettingen, Germany), was used in this study. The alga cells were grown in a 250 mL flask containing 100 mL ESP Ag medium [40] at 22 °C under continuous illumination of 25 μmol photon m^−2^s^−1^ in an incubator.

For the stress treatment to induce astaxanthin production, a final concentration of 45 mM sodium acetate was added into cultures when alga cells reached to the logarithmic phase (OD_730_ = 0.8), along with the exposure of continuous high light (irradiance at 500 μmol photon m^−2^s^−1^). To maximize the transcripts in the following SMRT sequencing, alga samples were harvested from the control (0 h) and at the time points of 1.5 h, 3 h, 6 h, 9 h, 12 h, 24 h, and 48 h after treatment. Alga cells were collected by centrifugation at 10,000× *g* for 5 min and frozen immediately by liquid nitrogen. Samples were stored at −80 °C until use.

### 4.2. Discovery, Isolation, and Sequencing of HpGGPPS Genes

Equal amounts of algae cells collected from different time points of stress treatment were pooled together for the total RNA extraction and sent to Gene Denovo Biotechnology Co. (Guangzhou, China) for SMRT sequencing to get the Iso-Seq data. To obtain *GGPPS* paralogs in *H. pluvialis*, a local BlastN against the Iso-Seq data was performed, using three reported *GGPPS* genes of *H. pluvialis* deposited in NCBI (Genbank accession no. KX236181, KX236182, and KX236183). To clone *HpGGPPS* candidates, PCR was performed using gene specific primers (Table 1) designed using the target nucleotide sequences from Iso-Seq data. The cDNA template for PCR was from the total RNA that was used for SMRT sequencing. The PCR reaction was carried out in a 20 µL volume containing 1 µL cDNA, 4.0 µL 5× SuperFi buffer, 4.0 µL 5× SuperFi GC Enhancer, 1.6 µL dNTP mix (2.5 mM), 1.0 µL each forward and reverse primer (10 µM), 0.2 µL SuperFi DNA polymerase (Invitrogen Life technologies, Carlsbad, CA, USA), and 7.2 µL ddH_2_O. The PCR was implemented on the Bio-Rad T100 thermal cycler (Bio-Rad, Hercules, CA, USA) under following conditions: 98 °C for 2 min; 35 cycles of 98 °C for 10 s, 60 °C for 10 s, 72 °C for 1.5 min; and a final extension at 72 °C for 5 min. PCR products were run on 1.0% agarose gel and target fragments were purified by E.Z.N.A. Gel Extraction Kit (Omega Bio-tek Inc. Norcross, GA, USA) according to the manufacturer’s instruction. Purified PCR fragments were treated by A-Tailing Kit (Takara, Japan), ligated into pGEM-T easy vector (Promega, Madison, WI, USA), and transformed into *E. coli* Top10 chemically competent cell, as described in manufacturer’s instructions. Positive colonies were selected by the blue-white screening strategy on the LB agar plate containing ampicillin (50 μg/mL) and sent to Guangzhou IGE Biotechnology Ltd. (Guangzhou, China) for sequencing.

### 4.3. Sequences Analysis and Molecular Evolution of HpGGPPS Genes

The ORFs of obtained nucleotide sequences of *HpGGPPS* genes and corresponding deduced amino acids were predicted by EditSeq module of DNASTAR software (Lasergene, Madison, WI, USA). Multiple sequence alignment was conducted by MegAlign module of DNASTAR software to determine the sequence identity and divergence. By aligned with genome sequences of *H. pluvialis* (BIG Data Center GSA Database accession no. PRJCA000614) [20], the gene structure was predicted and the schematic diagram was displayed by Gene Structure Display Server 2.0 (http://gsds.cbi.pku.edu.cn/). The domain of deduced amino acid was predicted by SMART online at http://smart.embl-heidelberg.de/. Phylogenetic relationship was analyzed by MEGA7 software with the neighbor-joining method from 1000 bootstrap replicates [41].

### 4.4. Plasmids Construction and Heterologous Expression

**Figure 6:**
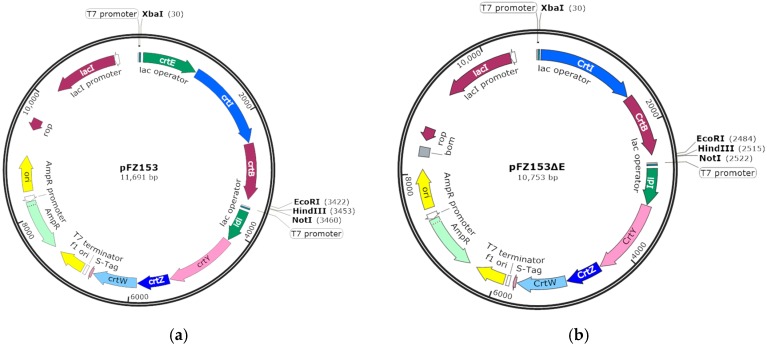
Structure of plasmid pFZ153 and pFZ153ΔE used to confer the function of *HpGGPPS* genes on astaxanthin biosynthesis in *E. coli* (BL21 DE3). The plasmid pFZ153 (**a**) was modified to construct the plasmid pFZ153ΔE (**b**) by removing the *CrtE* gene. In this study, *HpGGPPS* genes were inserted into the plasmid on *EcoR*I/*Hind*III or *EcoR*I/*Not*I site to complete the astaxanthin synthetic gene cluster. The *CrtE* gene from pFZ153 was served as the positive control.

The plasmid used for heterologous expression targeting gene functional analysis was adopted from pFZ153, a plasmid has astaxanthin synthetic gene cluster (*CrtE*, *CrtI*, *CrtB*, *idi*, *CrtY*, *CrtZ*, and *CrtW*) and produces astaxanthin in *E. coli* [42]. To investigate the function of *HpGGPPS* genes on astaxanthin synthesis, the pFZ153ΔE plasmid was constructed (Figure 6b). Firstly, *CrtE*, *CrtI*, and *CrtB* genes were removed from pFZ153 (Figure 6a) by *Xba*I/*EcoR*I digestion. Secondly, the coding sequence of CrtI/CrtB gene was amplified from pFZ153 plasmid using primers CrtIB-F2/CrtIB-R2 containing *Xba*I and *EcoR*I restriction sites, respectively. Finally, the gel-purified fragment of CrtI/CrtB gene was digested with *Xba*I/*EcoR*I restriction enzyme and then ligated with the pFZ153 fragment without *CrtE*, *CrtI*, and *CrtB* genes to form the new plasmid pFZ153ΔE.

To complete the astaxanthin synthetic gene cluster, *HpGGPPS* genes were inserted into pFZ153ΔE to form plasmids pFZ153ΔE/GGPPS. In detail, the coding sequences of *HpGGPPS* genes were amplified from the sequenced plasmids containing *HpGGPPS* genes, using primers listed in Table 1 containing either *EcoR*I/*Hind*III or *EcoR*I/*Not*I restriction sites. Similar as described above, the target PCR fragments were gel-purified and ligated into pFZ153ΔE plasmid in either *EcoR*I/*Hind*III or *EcoR*I/*Not*I sites. Additionally, the coding sequence of *CrtE* gene amplified from pFZ153 with primers CrtE-F1/CrtE-R1 harboring *EcoR*I/*Hind*III sites, was also ligated into pFZ153ΔE plasmid to construct the positive control (pFZ153ΔE/CrtE).

The procedures of PCR amplification using Platium SuperFi DNA polymerase and gel-purification using E.Z.N.A. Gel Extraction Kit are the same as described above. The ligation process was performed by T4 ligase (Promega, Madison, WI, USA), as described by the manual. *E. coli* Top10 was used as a host during plasmids construction, as described above. Restriction enzymes used in this study were purchased from Thermo Fisher Scientific Inc. (Waltham, MA, USA).

For the functional analysis of *HpGGPPS* genes, plasmids of pFZ153ΔE/GGPPS and pFZ153ΔE/CrtE were transferred into an expression host, *E. coli* strain BL21 (DE3). The transformed *E. coli* colonies were selected on the LB agar plate with ampicillin (100 µg/mL) and then cultured in 15 mL culture tubes containing 3 mL LB liquid media supplemented with 100 µg/mL ampicillin (LB+Amp100 medium) overnight. The aliquot of 0.5 mL was added into a 250 mL flask containing 100 mL LB+Amp100 medium. The flasks were incubated at 30 °C under dark with shaking (200 rpm) until OD_600_ reached 0.7–0.9, followed by adding IPTG to a final concentration of 0.1 mM. Three hours later, 50 mL of each culture was harvested and pigments were extracted as described below. For each plasmid, at least three independent *E. coli* colonies were selected for pigment extraction as replicates.

### 4.5. Pigment Extraction from E. coli and HPLC Analysis

The *E. coli* BL21 (DE3) cells expressing plasmids constructed in the study were harvested by centrifugation at 12,000× *g* for 5 min at 4 °C. The pellet was washed by ddH_2_O twice and dried using a freeze-dryer. Consequently, the pellet was crashed into powder, weighted, and resuspended in 1 mL acetone for pigment extraction. The suspension was then incubated at a water bath at 55 °C for 15 min with vigorous shaking every 5 min. The supernatant containing pigments was collected by centrifugation at 12,000× *g* for 10 min at 4 °C. The pigment was subsequently dried using a vacuum evaporator and dissolved in 0.5 mL solution A (methanol/isopropanol, 8:2, V:V) for the following HPLC analysis.

The HPLC assay was performed on a Series 1260 system (Agilent Technologies, Santa Clara, CA, USA). Pigments were separated at a flow rate of 0.6 mL/min on a Phenomenex Gemini-NX C18 column (5 μ, 150 × 3.0 mm, Phenomenex Inc., Aschaffenburg, Germany) using solution A and solution B (ddH_2_O). The gradient elution was 85% A, 15% B at 0 min, followed by linear gradient to 100% A at 10 min, maintained at 100% A to 12 min, returned to initial condition by 12.1 min, and re-equilibrated at initial condition by 18 min. The injection volume was 5 µL. Column temperature was maintained at 35 °C. The standard astaxanthin (Aladdin Chemistry Co. Ltd., Shanghai, China) was used to identify the target in the pigment. The detection was carried out by UV absorbance at 475 nm. To determine the concentration of astaxanthin in the testing sample, a calibration curve was constructed using a serial of plots with 500, 200, 100, 50, 20, and 10 µg/mL standard astaxanthin. The linearity between concentrations of astaxanthin and their corresponding peak areas was figured out to calculate the concentration of each analyte. The final astaxanthin productivity of each constructed plasmid was calculated by the formula, astaxanthin productivity = cal-con. × 0.5 mL/DW, where cal-con. refers to the calculated astaxanthin concentration in the analyte from the calibration curve, 0.5 mL comes from the final volume for each pigment extraction, DW comes from the amount of dried cells used for a pigment extraction. All organic solvents (acetone, methanol, and isopropanol) used in this study are HPLC grade.

### 4.6. Statistical Analysis

The data were processed by Pearson *t*-test to compare two means with replicates. The statistical significance was determined according to the *p* < 0.05.

## Figures and Tables

**Table 1:** Primers used in this study.

Primer ID	Sequences (5’–3’)	Used for
GGPP1F1	CACTGCCTATCCCCGTTTCCAATC	Cloning of HpGGPPS1-1 and HpGGPPS1-2
GGPP1R1	GCACCTGCTGACCCGCTCTG	
GGPP2F1	GGCGACGCGGGCAAATCAGT	Cloning of HpGGPPS2-1 and HpGGPPS2-2
GGPP2R1	AAGCGCCAGGGAATACCAAACATA	
GGPP3F1	GCTCTCTTCGCACTTCTTGG	Cloning of HpGGPPS3-1, HpGGPPS3-2, HpGGPPS3-3 and HpGGPPS3-4
GGPP3R1	TGATGCCTAGACAGCTCACTT	
fGGPP1F2	GTATCT*GAATTC*AAAAAATGATCCGAGCGATGCACA ^†^	Subcloning of HpGGPPS1-1
GGPP1R2	CATAGA*AAGCTT*TCAGTTCTTGCGGTATCCTA	
GGPP1F3	GTATCT*GAATTC*AAAAAATGATCCGAGCGATGCACA	Subcloning of HpGGPPS1-2
GGPP1R3	CATAGA*AAGCTT*TCAGTTCTTGCGGTACCCT	
GGPP2F2	GTATCT*GAATTC*AAAAAATGAGGGGCCTAGCGGGCAA	Subcloning of HpGGPPS2-1 and HpGGPPS2-2
GGPP2R2	CATAGA*GCGGCCGC*CTATTTCTTTCTGCTCAGGACTC	
GGPP3F2	GTATCT*GAATTC*AAAAAATGGTATCGGATGTGATGCAAG	Subcloning of HpGGPPS3-1
GGPP3R2	CATAGA*AAGCTT*TCACTTGCAGCGCTTGTAAATC	
GGPP3F3	GTATCT*GAATTC*AAAAAATGGTATCGGATGTGATGCAAG	Subcloning of HpGGPPS3-2, HpGGPPS3-3, and HpGGPPS3-4
GGPP3R3	CATAGA*AAGCTT*TCACTTGCAGCGCTTGTAAATC	
CrtIB-F2	GTATCT*TCTAGA*GTAAGGATCCTAACATGAAACCGACCACGGTGA	Subcloning of CrtIB
CrtIB-R2	CATAGA*GAATTC*ATGTCGACAAGTTACAGCGGACGTTGCCAC	
CrtE-F1	GTATCT*GAATTC*ATACCATGACCGTGTGTGCGAA	Subcloning of CrtE
CrtE-R1	ATAGA*AAGCTT*TCCTTTACGACACCGCTGCCA	

^†^ Restriction enzyme sites are formatted in italics.

## Supplementary Materials

The following are available online at https://www.mdpi.com/1660-3397/17/12/696/s1, Table S1: Sequence characterization of *HpGGPPS* genes. Figure S1. Sequence similarity and divergence of eight *HpGGPPS* genes in the coding region. The alignment was performed by Clustal W using MegAlign module in the DNASTAR software. Figure S2. The HPLC chromatograms of pigments extracted from transformed *E. coli* cells. (a) the profile of standard astaxanthin purchased from Aladdin Chemistry Co. Ltd (Shanghai, China); (b) an representative profile of the extracted pigment from *E. coli* harboring *HpGGPP* genes and *CrtE* gene.

## References

[1] Grand View Research Astaxanthin Market Analysis by Source (Natural [Yeast, Krill/Shrimp, Microalgae] And Synthetic), by Product (Dried Biomass/Powder, Oil, Soft gels, Liquid), by Application, and Segment Forecasts, 2018–2025. https://www.grandviewresearch.com/industry-analysis/global-astaxanthin-market.

[2] Higuera-ciapara I., Felix-valenzuela L., Goycoolea F.M. (2006). Astaxanthin: A review of its chemistry and applications. Crit. Rev. Food Sci. Nutr..

[3] Krause W., Henrich K., Paust J., Ernst H. Preparation of Astaxanthin. https://www.google.com/patents/US5654488.

[4] Koller M., Muhr A., Braunegg G. (2014). Microalgae as versatile cellular factories for valued products. Algal Res..

[5] Lorenz R.T., Cysewski G.R. (2000). Commercial potential for *Haematococcus microalgae* as a natural source of astaxanthin. Trends Biotechnol..

[6] McGarvey D.J., Croteau R. (1995). Terpenoid metabolism. Plant Cell.

[7] Shah M.M.R., Yuanmei L., Cheng J.J., Maurycy D. (2016). Astaxanthin-producing green microalga *Haematococcus pluvialis*: From single cell to high value commercial products. Front. Plant Sci..

[8] Rohmer M., Knani M., Simonin P., Sutter B., Sahm H. (1993). Isoprenoid biosynthesis in bacteria: A novel pathway for the early steps leading to isopentenyl diphosphate. Biochem. J..

[9] Liang C., Zhang W., Zhang X., Fan X., Xu D., Ye N., Yang Q. (2015). Isolation and expression analyses of methyl-d-erythritol 4-phosphate (MEP) pathway genes from *Haematococcus pluvialis*. J. Appl. Phycol..

[10] Sun Z., Cunningham F.X., Gantt E. (1998). Differential expression of two isopentenyl pyrophosphate isomerases and enhanced carotenoid accumulation in a unicellular chlorophyte. Proc. Natl. Acad. Sci. USA.

[11] Hoeffler J.F., Hemmerlin A., Grosdemange-Billiard C., Bach T.J., Rohmer M. (2002). Isoprenoid biosynthesis in higher plants and in *Escherichia coli*: On the branching in the methylerythritol phosphate pathway and the independent biosynthesis of isopentenyl diphosphate and dimethylallyl diphosphate. Biochem. J..

[12] Gwak Y., Hwang Y.S., Wang B., Kim M., Jeong J., Lee C.G., Jin E. (2014). Comparative analyses of lipidomes and transcriptomes reveal a concerted action of multiple defensive systems against photo oxidative stress in *Haematococcus pluvialis*. J. Exp. Bot..

[13] Britton G., Young A., Britton G. (1993). Biosynthesis of carotenoids. Carotenoids in Photosynthesis.

[14] Mende K., Homann V., Tudzynski B. (1997). The geranylgeranyl diphosphate synthase gene of *Gibberella fujikuroi*: Isolation and expression. Mol. Gen. Genet..

[15] Kainou T., Kawamura K., Tanaka K., Matsuda H., Kawamukai M. (1999). Identification of the *GGPS1* genes encoding geranylgeranyl diphosphate synthases from mouse and human. Biochim. Biophys. Acta..

[16] Coman D., Altenhoff A., Zoller S., Gruissem W., Vranová E. (2014). Distinct evolutionary strategies in the GGPPS family from plants. Front. Plant Sci..

[17] Merchant S.S., Prochnik S.E., Vallon O., Harris E.H., Karpowicz S.J., Witman G.B. (2007). The *Chlamydomonas* genome reveals the evolution of key animal and plant functions. Science.

[18] Gonzalez-Garay M.L., Wu J. (2016). Introduction to isoform sequencing using pacific biosciences technology (Iso-Seq). Transcriptomics and Gene Regulation.

[19] Abdelghany S.E., Hamilton M., Jacobi J.L., Ngam P., Devitt N., Schilkey F., Reddy A.S. (2016). A survey of the sorghum transcriptome using single-molecule long reads. Nat. Commun..

[20] Luo Q., Bian C., Tao M., Huang Y., Zheng Y., Lv Y., Xu J. (2019). Genome and transcriptome sequencing of the astaxanthin-producing green microalga, *Haematococcus pluvialis*. Genome Biol. Evol..

[21] Wayama M., Ota S., Matsuura H., Nango N., Hirata A., Kawano S. (2013). Three-dimensional ultra structural study of oil and astaxanthin accumulation during encystment in the green alga *Haematococcus pluvialis*. PLoS ONE.

[22] Capelli B., Bagchi D., Cysewski G.R. (2013). Synthetic astaxanthin is significantly inferior to algal-based astaxanthin as an antioxidant and may not be suitable as a human nutraceutical supplement. Nutrafoods.

[23] Linden H. (1999). Carotenoid hydroxylase from *Haematococcus pluvialis*: cDNA sequence, regulation and functional complementation. Biochim. Biophys. Acta Gene Struct. Expr..

[24] Lotan T., Hirschberg J. (1995). Cloning and expression in *Escherichia coli* of the gene coding β-C-4-oxygenase, that converts β-carotene to the ketocarotenoid canthaxanthin in *Haematococcus pluvialis*. FEBS Lett..

[25] Kajiwara S., Kakizono T., Saito T., Saito T., Kondo K., Ohtani T., Nishio N., Misawa N. (1995). Isolation and functional identification of a novel cDNA for astaxanthin biosynthesis from *Haematococcus pluvialis*, and astaxanthin synthesis in *Escherichia coli*. Plant Mol. Biol..

[26] Huang J.C., Chen F., Sandmann G. (2006). Stress-related differential expression of multiple β-carotene ketolase genes in the unicellular green alga *Haematococcus pluvialis*. J. Biotech..

[27] Ye L., Zhu X., Wu T., Wang W., Zhao D., Bi C., Zhang X. (2018). Optimizing the localization of astaxanthin enzymes for improved productivity. Biotechnol. Biofuels.

[28] Henke N.A., Wendisch V.F. (2019). Improved astaxanthin production with *Corynebcterium glutamicum* by application of a membrane fusion protein. Mar. Drugs.

[29] Wu Y., Yan P., Liu X., Wang Z., Tang Y.J., Chen T., Zhao X. (2019). Combinatorial expression of different β-carotene hydroxylases and ketolases in *Escherichia coli* for increased astaxanthin production. J. Ind. Microbiol. Biotechnol..

[30] Ruiz-Sola M.A., Coman D., Beck G., Barja M., Colinas M., Graf A., Gruissem W. (2016). *Arabidopsis* GERANYLGERANYL DIPHOSPHATE SYNTHASE 11 is a hub isozyme required for the production of most photosynthesis-related isoprenoids. New Phytol..

[31] Liang P.H., Ko T.P., Wang A.H.J. (2002). Structure, mechanism and function of prenyltransferases. Eur. J. Biochem..

[32] Tholl D., Kish C.M., Orlova I., Sherman D., Gershenzon J., Pichersky E., Dudareva N. (2004). Formation of monoterpenes in *Antirrhinum majus* and *Clarkia breweri* flowers involves heterodimeric geranyldiphosphate synthases. Plant Cell.

[33] Beck G., Coman D., Herren E., Ruiz-Sola M.Á., Rodríguez-Concepción M., Gruissem W., Vranová E. (2013). Characterization of the GGPP synthase gene family in *Arabidopsis thaliana*. Plant Mol. Biol..

[34] Hsieh F.L., Chang T.H., Ko T.P., Wang A.H.J. (2011). Structure and mechanism of an *Arabidopsis* medium/long-chain-length prenylpyrophosphate synthase. Plant Physiol..

[35] Lao Y.M., Jin H., Zhou J., Zhang H.J., Zhu X.S., Cai Z.H. (2018). A novel hydrolytic activity of tri-functional geranylgeranyl pyrophosphate synthase in *Haematococcus pluvialis*. Plant Cell Physiol..

[36] Wang J., Lin H., Su P., Chen T., Guo J., Gao W., Huang L.Q. (2019). Molecular cloning and functional characterization of multiple geranylgeranyl pyrophosphate synthase (ApGGPPS) from *Andrographis paniculata*. Plant Cell Rep..

[37] Kane J.F. (1995). Effects of rare codon clusters on high-level expression of heterologous proteins in *Escherichia coli*. Curr. Opin. Biotechnol..

[38] Gustafsson C., Govindarajan S., Minshull J. (2004). Codon bias and heterologous protein expression. Trends Biotechnol..

[39] Gao Z., Li Y., Wu G., Li G., Sun H., Deng S., Zhang X. (2015). Transcriptome analysis in *Haematococcus pluvialis*: Astaxanthin induction by salicylic acid (SA) and jasmonic acid (JA). PLoS ONE.

[40] Zheng Y., Li Z., Tao M., Li J., Hu Z. (2017). Effects of selenite on green microalga *Haematococcus pluvialis*: Bioaccumulation of selenium and enhancement of astaxanthin production. Aquat. Toxicol..

[41] Kumar S., Stecher G., Tamura K. (2016). MEGA7: Molecular Evolutionary Genetics Analysis version 7.0 for bigger datasets. Mol. Biol. Evol..

[42] Ma T., Zhou Y., Li X., Zhu F., Cheng Y., Liu Y., Liu T. (2016). Genome mining of astaxanthin biosynthetic genes from Sphingomonas sp. ATCC 55669 for heterologous overproduction in *Escherichia coli*. Biotechnol. J..

