# Supplementary material for: Discovery of Geranylgeranyl Pyrophosphate Synthase (GGPPS) Paralogs from Haematococcus pluvialis Based on Iso-Seq Analysis and Their Function on Astaxanthin Biosynthesis
Source: Mar Drugs. 2019 Dec 12;17(12):696. doi: 10.3390/md17120696 (PMC6950171; doi:10.3390/md17120696)
Supplement: Supplementary file 1 [file marinedrugs-17-00696-s001.pdf]

Supplementary Materials:

Supplemental Table S1. Sequence characterization of *HpGGPPS* genes

Gene	Accession#	Length (bp)	CDS (bp)	Putative amino acids (KDa)
HpGGPPS1-1	MN689792	1260	1017	36.45
HpGGPPS1-2	MN689793	1249	1017	36.43
HpGGPPS2-1	MN689794	1448	1272	45.75
HpGGPPS2-2	MN689795	1455	1272	45.71
HpGGPPS3-1	MN689796	1128	1077	40.71
HpGGPPS3-2	MN689797	1074	1023	38.77
HpGGPPS3-3	MN689798	1128	1077	40.76
HpGGPPS3-4	MN689799	1011	960	36.27

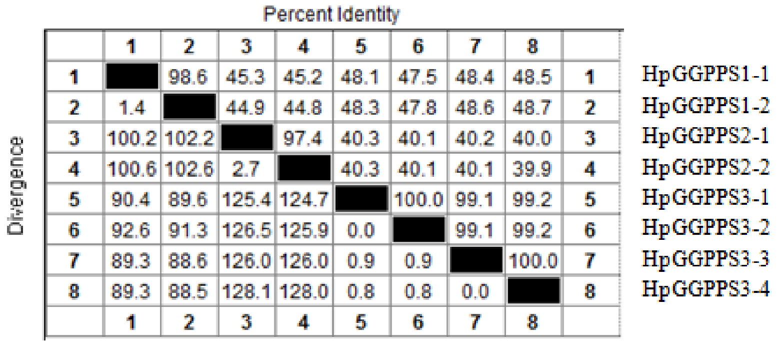

Percent Identity (upper triangle) / Divergence (lower triangle)

	1	2	3	4	5	6	7	8		
1		98.6	45.3	45.2	48.1	47.5	48.4	48.5	1	HpGGPPS1-1
2	1.4		44.9	44.8	48.3	47.8	48.6	48.7	2	HpGGPPS1-2
3	100.2	102.2		97.4	40.3	40.1	40.2	40.0	3	HpGGPPS2-1
4	100.6	102.6	2.7		40.3	40.1	40.1	39.9	4	HpGGPPS2-2
5	90.4	89.6	125.4	124.7		100.0	99.1	99.2	5	HpGGPPS3-1
6	92.6	91.3	126.5	125.9	0.0		99.1	99.2	6	HpGGPPS3-2
7	89.3	88.6	126.0	126.0	0.9	0.9		100.0	7	HpGGPPS3-3
8	89.3	88.5	128.1	128.0	0.8	0.8	0.0		8	HpGGPPS3-4
	1	2	3	4	5	6	7	8		

Supplemental Figure S1. Sequence similarity and divergence of eight HpGGPPS genes in the coding region. The alignment was performed by Clustal W using MegAlign module in the DNASTAR software.

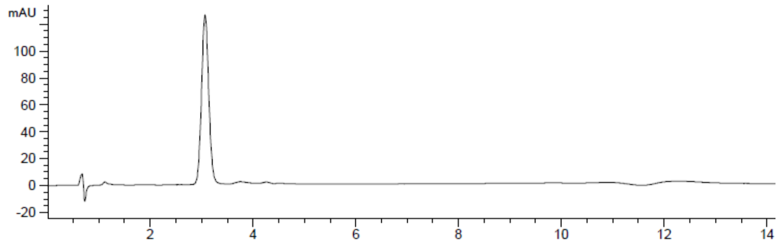

(a)

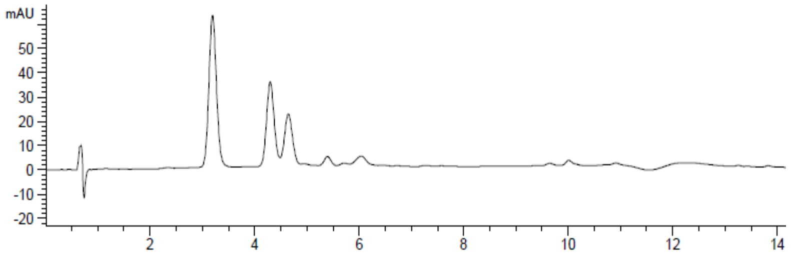

(b)

Supplemental FigureS2. The HPLC chromatograms of pigments extracted from transformed *E. coli* cells. (a) the profile of standard astaxanthin purchased from

Aladdin Chemistry Co. Ltd (Shanghai, China); (b) an representative profile of the extracted pigment from *E. coli* harboring HpGGPP genes and CrtE gene.
